# Do oxytocin neurones affect feeding?

**DOI:** 10.1111/jne.13035

**Published:** 2021-09-08

**Authors:** Amy A. Worth, Simon M. Luckman

**Affiliations:** ^1^ Faculty of Biology, Medicine and Health University of Manchester Manchester UK

## Abstract

There has been a long history of research on the effects of oxytocin on feeding behaviour. The classic‐held view is that the neurohormone is anorexigenic at least in rodents, although the data for humans are not so clear cut. Likewise, a physiological role for oxytocin is disputed. Thus, although pharmacological, anatomical and physiological data suggest oxytocin may have a function in satiety signalling, this view is not supported by the latest research using the genetic recording and manipulation of oxytocin neurones. Here, we avoid a discussion of the pharmacological effects of oxytocin and examine evidence, from both sides of the argument, concerning whether the endogenous oxytocin system has a role in the regulation of normal feeding.

There is little dispute that the neurohormone, oxytocin, if administered centrally or systemically, can affect food intake in rodents. A meta‐analysis of the literature published up to 2018 concluded that exogenous oxytocin decreases the amount of food eaten by rats or mice, whereas effects in humans were not supported by the analysis.^1^ For example, Leng and Ludwig^2^ question the interpretation of experiments involving industrial doses of oxytocin (“several pituitaries worth”) to humans nasally. Accordingly, whether oxytocin might be developed as a therapeutic treatment for obesity is controversial, and not a topic for this short commentary; for a recent review, see McCormack et al.^3^ Although there is evidence that pharmacologically, oxytocin can reduce food intake, the question of whether oxytocin and/or oxytocin (Oxt) neurones have a significant role in the normal, physiological regulation of appetite has long provoked an extreme polarisation of views, which we examine here.

Many of us have been jobbing neuroendocrinologists during a time when the standard tool for determining the physiological importance of a messenger was to knock it (or its receptor) out in the germline of laboratory mice. We bestow offerings to the gods of translation if our very expensive mice turn out to have the phenotype we hoped for (*Oxt* knockout mice cannot suckle their young), but we beg for mercy from the gods of redundancy when they do not (otherwise, these mice exhibit normal birth and maternal behaviour).^4^ Thankfully, for the champions of behavioural neuroendocrinology, mice lacking oxytocin do have deficits in social recognition and altered aggressive behaviour. Some assurance for a role in metabolism arrived when it was discovered that mice lacking the oxytocin receptor exhibit late‐onset obesity.^5^ However, neither *Oxt*, nor *Oxtr* knockout mice are hyperphagic (although the former have an increased preference for sweet solutions and the latter have larger meal size) and, instead, their phenotypes appear to be more dependent on a decrease in sympathetic tone to adipose tissues.^5^, ^6^ Does the phenotype of these two germline knockout mouse models fit with what is known from basic pharmacology? Central administration of oxytocin reduces body weight in animal models that are otherwise resistant to leptin (either because of their diet‐induced obesity or as a result of defective leptin signalling), with a concomitant improvement in glucose tolerance and insulin sensitivity.^7^, ^8^, ^9^, ^10^, ^11^, ^12^ Although it is postulated that the weight‐reducing effects are dependent on decreased eating, this does not prove a physiological role because pharmacotreatment is likely to affect a number of parameters that will impact on feeding either directly or indirectly.^13^, ^14^, ^15^ Furthermore, oxytocin administration to pair‐fed animals suggests there are additional effects on energy expenditure and on adipose tissue lipolysis.^8^, ^10^, ^11^, ^16^, ^17^ Some mismatch between pharmacology and embryonic knockout models is nothing unusual and might be explained by redundancy either within Oxt neurone function itself or within more widespread appetite‐regulating circuits in the brain. However, as is often the case, no absolute evidence for either has yet been provided.

There are other developmental models which do point towards an important role for Oxt neurones in regulating food intake. Both Prader‐Willi syndrome in human patients^18^, ^19^ and haploinsufficiency of the transcription factor *Sim1* in mice^20^ are correlated with low numbers of Oxt neurone, hyperphagia and obesity. Although it can be argued that neither model involves the selective deletion of Oxt neurones, the phenotype of both PWS humans and *Sim1* ^+/−^ mice can be reversed by oxytocin replacement.^21^, ^22^ Experimental models of Oxt neurone deficiency also exist. Cre‐dependent, diptheria toxin ablation of all adult oxytocin‐containing cells produces a complex phenotype.^23^ When on normal chow, the lesioned mice have the same body weight, food intake and energy expenditure as intact littermates. However, after changing to a high‐fat diet, male but not female lesioned mice show later‐onset obesity, again with no change in food intake, but with decreases in energy expenditure. Wu et al.^23^ rightly point out that Oxt neurones are not an absolute requirement for the regulation of body weight, although they acknowledge that there is precedent for rapid compensatory responses by redundant, central metabolic pathways. One telling observation is that mice with ablated Oxt neurones show an attenuated response to the acute anorectic effect of exogenous leptin. This is important because another group reports that, in a similar model, adult mice with ablation of Oxt neurones do not have altered cumulative food intake, although they increase sucrose consumption and do not respond to the acute anorectic effect of exogenous cholecystokinin.^24^ Surprisingly, for both these studies in which adult Oxt neurones are ablated with diptheria toxin, food intake was measured per week and no actual observations of daily intake or of meal patterning were reported, despite the indications that mice have attenuated responses to acute anorectic signals.

Our take‐home message from genetic models, which disrupt the expression of *Oxt* and *Oxtr*, or indeed kill Oxt neurones, is that the oxytocin signalling system is not indispensable. However, it might be imprudent to say that the system does not play a physiological role in regulating food intake. The lack of an obvious and consistent intake phenotype may reflect redundancy in the circuitry that is needed to control such a fundamental requirement for life. Life cannot exist without energy intake, although how much we eat during individual meals is not critical for survival. By comparison, measurements of energy expenditure and lipid metabolism in these models do not indicate redundancy. Although the evidence is not complete, locomotor activity and basal metabolic rate are not obviously affected, whereas brown adipose‐mediated adaptive thermogenesis and white adipose accretion are invariably impacted. Could this be because the evolutionary pressure to evolve redundancy in diet‐induced or non‐shivering, cold‐induced adaptive thermogenesis has not been so great? In short, rodents have the ability to adapt their diets, meal patterns and ways in which to keep warm. Furthermore, if there is redundancy, it appears to be more prevalent in female compared to male rodents (in *Oxtr* knockout mice and Oxt neurone‐lesioned mice, males but not females display late‐onset obesity). Could this redundancy in the female be because at certain times in a reproductive life, her oxytocin system needs to be otherwise employed?

Certainly, the most promising route forward to understanding normal physiology will be, wherever possible, to manipulate the fully differentiated, adult system. Vesicular release from hypothalamic Oxt neurones is negatively regulated by synaptagamin 4.^9^ Although *Syt4* is not expressed exclusively in Oxt neurones, it is strongly enriched in these cells. It is thus interesting that lentiviral over expression of *Syt4* in adult Oxt neurones in the paraventricular nucleus of the hypothalamus (Oxt^PVH^) reduces oxytocin secretion in vitro and leads to an obese phenotype in vivo. Zhang et al.^9^ backed up their conviction regarding a role for oxytocin in metabolic regulation by also reporting that either knockdown of *Oxt* in adult PVH neurones or central administration of Oxtr antagonist increased food intake and body weight. Thus, they have provided loss‐of‐function evidence for a role for oxytocin in adult feeding. Alternative, selective targeting of Oxt neurones, without killing them, is possible through the use of *Oxt*‐Cre mice and viral delivery of Cre‐dependent tetanus toxin to inhibit synaptic transmitter release. Li et al.^25^ followed the long‐term body weight and food intake of mice with tetanus toxin expressed exclusively in paraventricular neurones with the Cre drivers *Oxt*, glucagon‐like peptide receptor 1 (*Glp1r*) or melanocortin receptor 4 (*Mc4r*). Although disabling either *Glp1r*‐Cre or *Mc4r*‐Cre neurones lead to hyperphagia and increased weight gain, targeting *Oxt*‐Cre neurones did not. Unfortunately, as for the diptheria toxin studies, a similar caveat applies because only weekly food intake data are presented.

Sutton et al.^26^ instead used a gain‐of‐function approach with the *Oxt*‐Cre model. They found that approximately one‐fifth of *Sim1*‐Cre::GFP neurones contain nitric oxide synthase (NOS1) immunoreactivity, and approximately one‐fifth of PVH *Nos1*‐Cre::GFP neurones contain oxytocin immunoreactivity (note: these are estimations because the proportions of overlap will be dependent on technical considerations, such as potential over‐expression of green fluorescent protein in adult neurones that might not normally express these drivers). *Sim*‐Cre, *Nos1*‐Cre or *Oxt*‐Cre mice were transduced by bilateral PVH viral delivery of an excitatory designer receptor, hM3Dq. Mice were prevented from feeding during the 9‐h period before lights out, and then food was returned and they were injected with vehicle. The following day, the same procedure (including food withdrawal) was followed, but mice were instead injected with the designer drug, CNO. Activation of *Sim*‐Cre or *Nos1*‐Cre PVH neurones, but not *Oxt*‐Cre PVH neurones, decreased subsequent night‐time food intake. Likewise, in another study using channel rhodopsin (ChR2)‐mediated optogenetic stimulation of adult Oxt^PVH^ neurones, no acute suppression of refeeding was recorded in 24 h‐fasted mice.^27^ This would appear to put a nail in the coffin for the role of Oxt^PVH^ neurones with respect to affecting fast‐induced food intake. However, there are some noteworthy points related to these studies. First, again meal patterns were not recorded and, second, no indication was given to the number of neurones actually transduced or activated. Notably, in two independent experiments,^26^ the activation of *Oxt*‐Cre PVH neurones increased oxygen consumption in one cohort of mice, but not in another cohort. Thus, it is extremely important that the efficacy of the genetic manipulation is validated and, if possible, correlated with the measured experimental effect. Third, in both studies, the mice had been food deprived and so other satiation signals would not have been present. This may be of particular note because an important function of oxytocin neurones may be to increase sensitivity to other satiety‐related signals.^28^, ^29^

Consideration needs to be given to the potential meal‐related stimuli, which might engage the oxytocin system. Most importantly, activation of Oxt neurones in both the PVH and the supraoptic hypothalamic nuclei by naturally occurring, meal‐related signals has been demonstrated using Fos activity mapping, including after fast‐induced re‐feeding^30^, ^31^ or following a sucrose meal.^32^, ^33^ Moreover, artificial activation can also provide important information, although this needs to be viewed cautiously because Oxt neurones are sensitive to both nausea and stress, which may comprise uncontrolled variables with certain experimental manipulations. That accepted, meal‐related activation is mimicked by the gut‐produced satiety hormone, cholecystokinin, and by gastric distension.^13^, ^34^, ^35^, ^36^ Likewise, “exogenous” administration of other metabolic hormones (leptin, fibroblast growth factor FGF21, melanocortin)^28^, ^29^, ^37^, ^38^ or nutrients (glucose, leucine, oleoylethanolamide)^39^, ^40^, ^41^ also activates Oxt neurones. Often, the anorexia induced by these artificial stimuli is blocked by antagonism of the oxytocin receptor.

Despite the poor temporal resolution of using the induction of c‐*fos* mRNA/Fos protein as indicators of cellular activation, there is a convincing body of evidence that oxytocin neurones respond to different signals of ingestion, nutrition and energy status. Real‐time, electrical recording from identified Oxt neurones in relation to comparative stimuli has been limited to anaesthetised rats; Oxt neurones have reduced electrical activity in fasted rats, although they do respond robustly to systemic leptin^42^ or cholecystokinin.^34^, ^35^ Therefore, it is surprising that, with the advent of in vivo calcium imaging, no evidence has emerged for Oxt neurone activation in response to food‐related stimuli. Li et al.^25^ used fibre photometry to record from different PVH neurone populations using the genetically encoded calcium indicator, GCaMP6s. *Glp1r*‐Cre and corticotrophin‐releasing hormone (*Crh*)‐Cre neurones responded to the presentation of food in fasted mice, whereas this was not the case for *Mc4r*‐Cre or *Oxt*‐Cre neurones. Resendez et al.^43^ used two‐photon imaging of Oxt^PVH^ neurones in awake, but head‐fixed mice. There is a striking heterogeneity in the response of neurones to social versus non‐social stimuli (anaesthetised conspecific or plastic bottle), and no immediate response to licking sucrose from a spout. In these two published studies, calcium recordings were made for 5 min or 3 s after presentation of the stimulus, respectively, and so focus on food sensation rather than food ingestion.

Oxt neurones are placed anatomically to respond to appetite‐related signals. Two specific neuronal inputs, one hypothalamic and one extra‐hypothalamic, have received much interest and should be considered in their physiological context: states of hunger and satiety, respectively. Neurones in the hypothalamic arcuate nucleus, which colocalise agouti‐related peptide (AgRP), neuropeptide Y and GABA, are active during the state of hunger, and make direct inhibitory synaptic contacts with Oxt^PVH^ neurones, as shown unequivocally by channel rhodopsin‐assisted circuit mapping (CRACM).^27^ AgRP^ARC^ have an essential role in the normal appetite regulation of adult mice^44^ and the artificial stimulation of these neurones causes robust feeding.^45^, ^46^ Atasoy et al.^27^ demonstrated the importance of Oxt neurones to this function because co‐stimulation of Oxt^PVH^ neurones blocked the effect of inhibitory AgRP^ARC^ terminals in the PVH to cause feeding. Interestingly, however, they also found that bilateral stimulation of Oxt^PVH^ neurones alone did not reduce re‐feeding in food‐deprived mice. Indeed, the role for Oxt^PVH^ downstream to AgRP^ARC^‐induced consumption is directly contradicted by another group. Garfield et al.^47^ also used CRACM to investigate AgRP^ARC^ connections with the PVH and failed to identify a direct projection to Oxt^PVH^ neurones, instead highlighting projections to non‐oxytocin, *Mc4r*‐containing neurones. It is difficult to determine which, if either, of these two groups is totally correct in their assessments of the role of Oxt^PVH^ neurones and whether or not they lie downstream of AgRP^ARC^. Because most evidence suggests Oxt neurones are activated by the normal consumption of food and, generally, oxytocin is considered to be anorectic, it has been hypothesised that there is a role in either satiation or satiety (which determine the length of a meal and the inter‐meal interval, respectively). Cholecystokinin, released primarily by the duodenum after a meal, activates sensory afferents of the vagus nerve which terminate in the brainstem, namely the nucleus of the tractus solitarius (NTS). A variety of short, vago‐vagal reflexes modulate gut function and, at the same time, NTS neurones co‐expressing noradrenaline and prolactin‐releasing peptide (PrRP) project directly to Oxt neurones in the hypothalamus.^31^, ^48^, ^49^, ^50^ Thus, a distinct satiation pathway, from the detection of nutrients in the gut to the secretion of oxytocin exists. The potential relevance of this particular pathway is borne out by the fact that the anorectic action of cholecystokinin is lost and mice become obese if they are missing PrRP from NTS neurones or if they lack the PrRP receptor.^50^, ^51^ A specific role for oxytocin itself in this satiation axis has yet to be confirmed, although, as observed previously, cholecystokinin has long been known to activate Oxt neurones and cause the secretion of the neurohormone. It must be noted, however, that injection of cholecystokinin does not recapitulate endogenous activation of nutrient‐induced satiety. Eating a meal does elicit the secretion of oxytocin into the circulation, although the injection of high doses of cholecystokinin, similar to other nauseous stimuli, induces much greater secretion.^13^

In turn, how might Oxt neurones affect appetite? It is hypothesised that Oxt^PVH^ neurones respond to anorectic gut signalling and influence gastric motility and food intake through a descending pathway to the dorsal vagal complex.^13^, ^14^, ^40^, ^52^, ^53^, ^54^, ^55^ Oxt^PVH^ neurones provide preautonomic innervation to activate cholinergic vagal motor neurones^56^, ^57^, ^58^ which, in turn, reduce gastric motility via a non‐adrenergic, non‐cholinergic pathway.^59^ A slowing of gastric emptying may be sufficient to induce meal termination. Alternatively, oxytocin might influence gut‐brain satiety signalling directly. In addition to being present on neurones of the motor nucleus of the vagus, Oxtr are also present on afferent sensory neurones of the vagus,^12^, ^60^, ^61^ as well as on second‐order neurones in the NTS.^62^ Leptin‐sensitive Oxt^PVH^ neurones project to the brainstem and decrease meal size by increasing the sensitivity of NTS neurones to satiety signals.^29^, ^62^ Injection of Oxtr antagonist into the fourth ventricle increases meal size, whereas lesions of hindbrain neurones with Oxtr reduce responses to meal signals.^62^, ^63^ Importantly, manipulation of brainstem signalling modifies meal patterning, without greatly altering daily food intake.

Alternatively, oxytocin may function in more rostral brain regions to affect appetitive circuits; either via direct axonal projections from (parvocellular) Oxt^PVH^ neurones or by local release of oxytocin from axon collaterals or the dendrites of magnocellular neurones.^64^, ^65^ A subpopulation of glutamatergic ARC neurones, some also containing pro‐opiomelanocortin, have been identified recently as containing Oxtr and which project directly back to the PVH to engage melanocortin‐receptive neurones.^66^ Interestingly, there is also strong anatomical and electrophysiological evidence for a direct projection of Oxt^PVH^ neurones to other forebrain structures, including the bed nucleus of the stria terminalis (BNST), ventral tegmental area (VTA) and nucleus accumbens (NAc).^67^, ^68^, ^69^, ^70^ The BNST is considered part of the “extended amygdala” and, thus, has been implicated in the neuroendocrine control of complex behaviours including stress, reward and appetite. The BNST exerts powerful control over motivational feeding, via GABAergic projections to the lateral hypothalamus (LH) and the VTA.^71^, ^72^ Hence, activation of BNST GABAergic projections stimulate food intake. Because Oxtr‐positive neurones in the BNST appear themselves to be GABAergic,^73^ oxytocin may cause local inhibition of the LH‐ and VTA‐projecting cells (similar to that occurring with an anxiolytic pathway utilising oxytocin in the amygdala).^70^ Injection of oxytocin into the VTA^74^ or NAc^75^ reduces sucrose intake and motivation to work for food. If given choices, *Oxt* knockout mice initiate more bouts of drinking sweet or non‐sweet carbohydrate, but not lipid emulsions,^76^, ^77^ suggesting a potential role in macronutrient choice. Indeed, administration of a brain‐penetrant Oxtr antagonist increases glucose ingestion.^78^ FGF21 is a putative upstream mediator that provides systemic input onto oxytocin neurones to modulate sucrose intake.^37^ Further work is required to determine the relative importance of oxytocin in the modulation of carobohydrate versus other macronutrient ingestion, as well as its role in food preference.

The evidence of a role for Oxt neurones in feeding behaviour has built up over the last 40 years but has been challenged recently following the introduction of the latest genetic tools. The ability to record or manipulate Oxt neurones selectively in behaving animals is game changing. However, there are still several important technical considerations, given that oxytocin influences so many factors typically leading to a cessation of food intake. Oxt neurones form a relatively small population, comprising approximately 4% of Sim1‐positive cells in the PVH^26^ or 2% of Glp1r/Mc4r^PVH^ neurones.^25^ Thus, although recording calcium dynamics in large or homogeneous populations is robust,^25^ the results from small or heterogeneous populations may be more problematic. Indeed, the one published work using two‐photon imaging of individual neurones, suggests that the Oxt^PVH^ population is very heterogeneous.^43^ Approximately, half of Oxt^PVH^ neurones increase intracellular calcium in response to a social cue and half reduce it. Also, of the neurones that respond positively to a social cue, half of these also respond to a non‐social cue, whereas the other half are inhibited. These studies concentrate on cell‐body dynamics, whereas calcium flux leading to the dendritic release of oxytocin may be important in some functions. Also of extreme importance is the time frame over which recordings are made because they should reflect the potential function of Oxt neurones in post‐ingestive regulation, rather than immediate sensory responses to food. To date, recordings are presented at most for the few minutes after presentation of food, whereas post‐ingestive/post‐absorptive signalling will take longer to kick in. Lastly, although care is taken to avoid stressing the experimental mice in these highly invasive experiments, fasting is itself a stress. Therefore, in experiments that utilise calcium imaging, it can be difficult to gauge the absolute activation state of neurones at baseline. For the reasons above, it would be helpful to investigate activity over the post‐ingestive phase and employ positive controls for Oxt neurone activation (which should be achievable with acute injections of cholecystokinin or hyperosmotic saline). Similar caution should also be applied to experiments that aim to artificially stimulate small populations of neurones. Thus, chemogenetic or optogenetic stimulations require supporting evidence that the neurones are being adequately activated and in a way that best models a physiological situation. It appears likely that Oxt neurones are modulatory and, thus, only affect food consumption under certain behavioural paradigms, such as when other satiety signals are present and other competing social signals are not. Although there is so much still to learn about the normal roles of Oxt‐containing cells, we should be careful before dismissing any function of these intriguing hypothalamic neurones.

## CONFLICT OF INTERESTS

The authors report no conflicts of interest.

## AUTHOR CONTRIBUTIONS

**Amy A. Worth:** Conceptualisation; Writing – original draft; Writing – review & editing. **Simon M. Luckman:** Conceptualisation; Writing – original draft; Writing – review & editing.

### PEER REVIEW

The peer review history for this article is available at https://publons.com/publon/10.1111/jne.13035.

## Data Availability

Data sharing not applicable to this article as no datasets were generated or analysed during the current study.
